# Incidence of perioperative sleep-disordered breathing in patients undergoing major surgery: a prospective cohort study

**DOI:** 10.1186/1754-9493-8-13

**Published:** 2014-03-13

**Authors:** Jens Roggenbach, Patrick Saur, Stefan Hofer, Thomas Bruckner, Michael Preusch, Remo Carbone, Andreas Walther

**Affiliations:** 1 Department of Anaesthesiology and Intensive Care Medicine, University Hospital, University of Heidelberg, Heidelberg, Germany; 2 Department of Medical Biometry and Informatics, University of Heidelberg, Heidelberg, Germany; 3 Department of Cardiology, Angiology, Pneumology, University Hospital, University of Heidelberg, Heidelberg, Germany; 4 Department of Anaesthesiology and Intensive Care Medicine, Katharinen-Hospital, Klinikum Stuttgart, Germany

**Keywords:** Obstructive sleep apnea, Major surgery, Sleep-disordered breathing, Postoperative hypoxemia

## Abstract

**Background:**

Major surgery might have a modulating effect on nocturnal breathing patterns. The incidence and course of perioperative sleep-disordered breathing in individuals without a previous diagnosis of obstructive sleep apnea has not been investigated sufficiently so far.

**Methods:**

In this study, polygraphic recordings have been obtained from 37 inpatients without a diagnosis of obstructive sleep apnea syndrome during the preoperative night before and six nights following major surgical procedures.

Eligible patients consenting to participate in this study underwent polygraphic recordings including four items (O_2_-saturation, pulse, nasal air flow and snoring) during the study period. Polygraphic data obtained from the postoperative recordings were compared to preoperative recordings.

**Results:**

Median (IQR *[range])* apnea-hypopnea-index (AHI) for the whole group was 6,0 (2,5 - 14,7 *[0–32,6]*) in the preoperative night and increased in the following six nights post surgery: second night: 5,6 (2,6-15,0 *[1,1 - 59,3]*); third night: 16,9 (5,6 - 38,8 *[2,9 - 64,3]*); fourth night: 11,6 (5,9 - 17,3 *[0,4 - 39,3]*); fifth night: 15,2 (5,7 - 22,2 *[0,2 - 55,5]*); sixth night: 22,5 (5,2 - 35,4 *[0,2 - 67,7]*). AHI-scores of the third to sixth night post surgery differed significantly from data observed in the preoperative night.

**Conclusion:**

A significant increase in the AHI occurred frequently after major surgical procedures in the late postoperative period. Sleep-disordered breathings in the late postoperative period deserve attention, as they potentially increase the risk of postoperative complications.

## Introduction

Sleep-disordered breathing (SDB) is a common disorder, with an estimated prevalence of 9% for women and 24% for middle-aged male adults. In the elderly, the prevalence is even higher [1-3]. It is characterized by intermittent cessation of normal respiratory patterns during sleep, most frequently due to a periodic loss of pharyngeal muscle tone with consecutive narrowing of the upper airway. In the most common form of SDB, the obstructive sleep apnea syndrome (OSAS), recurrent loss of airway patency causes intermittent hypoxemia and hypercapnia, which is associated with endogenous stress, sympathoadrenergic activation and recurrent arousals. Severe OSAS can lead to sleep fragmentation and interruption of the normal sleep architecture. In the perioperative setting OSAS has been associated with respiratory complications [4,5], myocardial ischemia [6], arrhythmias, postoperative delirium, bleeding and wound infections [7-10]. It is noteworthy that even though SDB and OSAS have been recognized as risk factors for perioperative complications, they mostly remain undiagnosed.

While the obstructive sleep apnea syndrome (OSAS) has been considered as a significant risk factor for perioperative morbidity, especially due to respiratory complications in the early perioperative episode, the dynamic process of postoperative sleep-associated respiratory disorders still remains to be elucidated in individuals without the diagnosis of OSAS. Since most individuals with OSAS are unaware of their disorder [11], it was the aim of this study to examine the course of SDB in the postoperative period in individuals undergoing major surgical procedures without a previous diagnosis of OSAS.

## Methods

This study was designed as a pilot trial, since literature provided only insufficient information on the postoperative course of SDB in individuals without previous diagnosis of OSAS. The institutional ethics committee of the university of Heidelberg approved the study. Between March and July 2010 written consent was obtained from inpatients undergoing major abdominal or urological surgery who were willing and eligible to participate in this study. Inclusion criteria were: age > 18 yrs, no previous diagnosis of sleep apnea, exclusion of any mental disability and a planned surgical procedure, requiring a postoperative in-hospital stay for at least six days.

Polygraphic recordings of nocturnal breathing disorders were performed with a portable polygraph (Mini-Screen 4, Heinen-Löwenstein, Bad Ems, Germany). The Mini-Screen 4 Polygraph measures heart rate and oxygen saturation with a pulse oxymeter and respiration and snoring via a nasal probe. Measurements were obtained from each participant from the night before surgery, and whenever possible, from the following six postoperative nights. Data on relevant coexisting diseases, permanent medication and administered analgesics and sedatives were collected for each patient. A check-up and adjustment of the polygraph was done every evening. Two trained physicians evaluated the polygraphic recordings. Sleep associated respiratory disorders were classified according to the American Academy of Sleep Medicine (AASM) criteria for the scoring of sleep and associated events [12]. According to the respiratory scoring rules published by the AASM an apnea was scored, when respiratory flow dropped by ≥ 90% of baseline for at least 10 seconds. A hypopnea was scored when nasal pressure signal excursions dropped by ≥ 50%, with a duration of at least 10 seconds, accompanied by a oxygen desaturation of at least 3% [12]. Exclusion criteria for nocturnal measurements were loss of the nasal probe or the pulse oxymeter during the night, or a recording time less than 6 hrs.

The apnea-hypopnea-index (AHI) expresses the mean number of apneas and hypopneas per hour sleep. In this study, a pre-operative AHI-Index < 5 was considered unsuspicious for OSAS.

Since this was a pilot trial, no data on normal pre- and postoperative AHI- values were available. In an a priori power analysis was performed with mean preoperative AHI- values (μ_1_), mean postoperative AHI-values (μ_2_) and the standard deviation (σ). Based on the assumptions μ_1_ = 5, μ_2_ = 10 and σ=5 a sample size of n = 10 was calculated with a significance level of p = 0.05 and a power of 0.8. Statistical analysis were performed with SPSS, Statistics pack 20.0 software (Chicago, IL, USA). The normality of data distribution was determined using the Kolmogorov-Smirnov test. Patient characteristics are expressed as mean and standard deviation for normally distributed data and as median, interquartile range (IQR) and range for non-normally distributed data. Postoperative measurements were compared with preoperative values using Boferroni adjusted Wilcoxon signed-rank tests. A value of p < 0,05 was considered significant.

## Results

Initally 70 patients consented to participate in the study. 19 Patients had to be excluded from the study, either due to insufficient polygraphic recordings preoperatively or during the nights following surgery. 14 patients refused further study participation after surgery. Finally, 37 Patients were included in this study. Patient characteristics are expressed in Table 1 and the type surgery is presented in Table 2. 15 patients (41%) of all patients consenting to participate, were admitted for prostate surgery. All other patient had major intraabdominal surgery (mainly pancreatic and small bowel surgery, colonic surgery and liver resection). Surgery was performed in all patients under general anesthesia. 13 patients had general anesthesia with neuraxial blockade via epidural catheter. Of which, 12 patients were admitted for visceral surgery. Postoperative pain therapy was performed either with continuous epidural anesthesia with ropivacaine (0.2%) or with i.v. opioid boluses and patient controlled analgesia using opioid boluses (piritramide) in all cases. All patients received either metamizol or non-steroidal anti-inflammatory drugs for supplementary pain therapy. After leaving the post-anaesthesia care unit, none of the examined individuals received supplemental oxygen during the evaluation period. None of the patients examined, spent more than one night in the post-anaesthesia care unit.

**Table 1:** Patient characteristics

Sex (m/f)	29/8
Age (years)- mean ± SD	60 ± 10
Height (cm)- mean ± SD	177,7 ± 8,9
Weight (Kg)- mean ± SD	84,6 ± 17,1
BMI- mean ± SD	26,6 ± 3,9
ASA-class (I/II/III)	2/24/11
NYHA-class > 0 (y/n)	5/32
Diabetes (y/n)	6/31
Coronary artery disease (y/n)	5/32
Chronic obstructive lung disease (y/n)	3/34
Arterial hypertension (y/n)	18/19
Renal insufficiency (y/n)	7/30
Duration of surgery (min ± SD)	234 ± 94

**Table 2:** Type of surgery performed in study sample

Pancreatic surgery	n = 5
Colorectal surgery	n = 5
Radical prostatectomy	n = 5
Laparoscopic prostatectomy	n = 10
Hemihepatectomy	n = 3
Peritonectomy	n = 2
Small bowel surgery	n = 4
Others	n = 3

No intraoperative complications were observed. During the postoperative period six patients (16.2%) had wound healing deficits and two patients had wound infections (5.4%). Furthermore, pulmonary embolism was diagnosed in two patients and one patient had refractory arterial hypertension.

**Figure 1:**
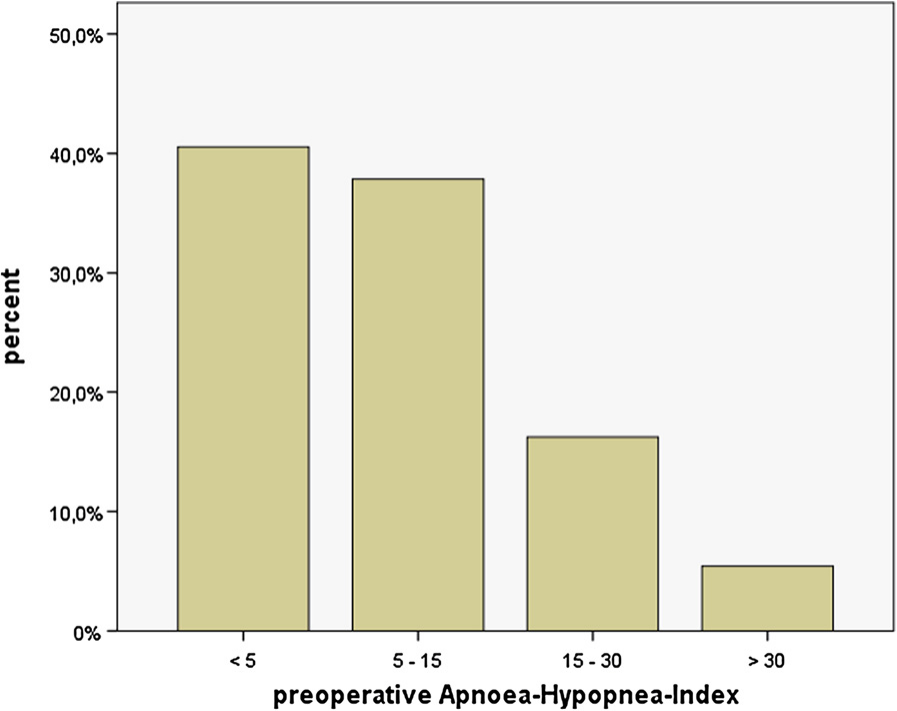
Prevalence and severity of SDB (expressed as AHI) in patients undergoing major surgery.

41% of all examined patients showed normal breathing patterns in the preoperative night. However, almost 22% of all patients had an AHI of 15 or higher (Figure 1).

While pre-surgery polygraphic recordings were obtained from all patients, only four patients continued their participation immediately the first night following surgery. According to patients self-evaluation the study dropout in the first night following surgery was mainly related to postoperative stress. Median (IQR *[range])* apnea-hypopnea-index (AHI) for the whole group was 6,0 (2,5 - 14,7 *[0–32,6]*) in the preoperative night and increased in the following six nights post surgery: second night: 5,6 (2,6-15,0 *[1,1 - 59,3]*); third night: 16,9 (5,6 - 38,8 *[2,9 - 64,3]*); fourth night: 11,6 (5,9 - 17,3 *[0,4 - 39,3]*); fifth night: 15,2 (5,7 - 22,2 *[0,2 - 55,5]*); sixth night: 22,5 (5,2 - 35,4 *[0,2 - 67,7]*) (Table 3). The AHI for the postoperative nights 3 to 6 differed significantly when compared to the preoperative data (preoperative AHI compared to AHI 3. night: p = 0,01; 4. night p = 0,045; 5. night p = 0,005; 6. night p = 0,04). The mean duration of apnoeic- and hypopnoeic episodes did not change significantly between the pre-op values and the following nights.

**Table 3:** AHI in the perioperative period: median (IQR [range]) in all examined individuals

	**All individuals (n = 37)**	**Sign. difference to preoperative values (Wilcoxon-Test - Bonferroni adjusted)**
Preoperative night (n = 37)	6,0 (2,6 - 14,7 [0–32,6])	
1. Postoperative night (n = 4)	4,6 (1,8 - 9,1 [1,2 - 10,2])	
2. Postoperative night (n = 18)	5,6 (2,6 - 15,0 [1,1 - 59,3])	n.s.
3. Postoperative night (n = 21)	16,9 (5,6 - 38,8 [2,9 - 64,3])	p = 0,01
4. Postoperative night (n = 28)	11,6 (5,9 - 17,4 [0,4 - 39,3])	p = 0,045
5. Postoperative night (n = 28)	15,2 (5,7 - 22,2 [0,2 - 55,5])	p = 0,005
6. Postoperative night (n = 11)	22,5 (5,2 - 35,4 [0,2 - 67,7])	p = 0,04

Frequent snoring was reported by 28 patients (76%), while only 9 patients (24%) complained of daytime sleepiness. Neither patients with self-reported snoring nor daytime sleepiness differed in their pre- and postoperative AHI-values from patients without these symptoms. No statistical difference in postoperative AHI values was observed, when comparing postoperative AHI values in patients with and without epidural analgesia in the subgroup of patients that underwent major visceral surgery. No correlation was found between the daily dosing of opioid-based analgesics and the AHI-values during the corresponding night. Sedative agents were given infrequently. Only three patients received benzodiazepines in the night before surgery.

## Discussion

In this pilot study we have observed a high prevalence of SDB and a significant increase in nocturnal AHI-values in surgical patients without a previous diagnosis of OSAS in the first six nights following the surgical procedure. Thus, postoperative SDB appears to be a common phenomenon, which can be found in numerous individuals and which is not restricted to patients with an already known diagnosis of sleep apnea. The severity of perioperative respiratory disturbances were not found to be different in individuals with and without the leading symptoms of OSAS, snoring and daytime sleepiness. Thus, screening for these symptoms to identify patients with undiagnosed OSAS might be of limited value.

The causes for the postoperative deterioration of SDB remain to be elucidated. It has been reported that perioperative analgetic therapy with opioids increases the risk for sleep related respiratory disturbances [13,14]. In this study however, we found no difference in the severity of postoperative SDB between patients with epidural analgesia and opioid based patient controlled analgesia. It has been shown that rostral fluid shift during recumbent position reduces upper-airway size and increases the upper airway collapsibility [15,16].

Due to perioperative fluid load, major abdominal surgery is regularly associated with significant fluid accumulation, which has to be eliminated after surgery. It appears reasonable, that perioperative volume retention might contribute to peripharyngeal soft tissue edema and thus, reduced airway patency. Furthermore, abundant literature suggests, that cytokines play an important role in sleep regulation. Especially the cytokines Interleukin-1 and Tumor-Necrosis-Factor, which are released after surgery, have been associated with sleep fragmentation, increased non-REM-sleep and suppression of REM-sleep [17-19]. Consistent with these observations, previous studies showed a suppression of REM and slow-wave sleep in the early postoperative period with a consecutive REM-rebound later on [20-22]. Increased and prolonged REM-Stages in the late postoperative period have been associated with a loss in pharyngeal musculature tone, predisposing to upper airway obstruction and thus, contributing to nocturnal desaturations [23,24].

As previously mentioned, the majority of OSAS remains undiagnosed in the general population [1,25]. Thus, it seems to be a frequent situation that the care-taking physician is confronted with patients suffering from undiagnosed OSAS. Notably, optimal treatment and surveillance of inpatients with OSAS undergoing surgery remains unclear. It has been suggested by the ASA task force on perioperative management of patients with obstructive sleep apnea that individuals with known or suspected OSAS should be monitored with pulse oximetry, until their oxygen saturations remain above 90% during sleep without supplemental oxygen [26]. However, it appears plausible, that sleep fragmentation and suppression of REM- and slow-wave-sleep might obscure the real incidence of sleep associated respiratory disorders in the early postoperative period. Therefore, after a fallacious uneventful first postoperative night, it might be a misconception to conclude that further monitoring might not be necessary. In this study nocturnal respiratory disturbances occurred primarily in the later postoperative period, when patients have been usually transferred to the regular ward and sufficient surveillance for hypoxemia is usually omitted. Thus, it is conceivable that postoperative sleep associated hypoxemic episodes usually remain unrecognized by professionals. Untreated hypoxemic events might increase postoperative stress and might consequently contribute to perioperative complications. It is worth mentioning that in a previous study on 206 patients with a diagnosis of OSAS that underwent ambulatory surgery, a high incidence of postoperative hypoxemia was found during the first night following ambulatory surgery. This however, was not associated with postoperative complications or unplanned hospital admission [27]. Thus, the meaning of sleep associated postoperative hypoxemia for postoperative complications deserves further investigation.

There are some limitations in the present study. Results of clinical observational studies can be biased by the individuals that are included in the study. It can not be excluded, that especially patients who were susceptible for symptoms of sleep apnea were willing to participate in this study. However, the observed prevalence of SDB in this study sample is comparable with observations from epidemiological studies [11,28].

In this trial, SDB was evaluated with polygraphs, not polysomnography, which might have overestimated the total sleeping time. It is noteworthy that various studies showed a high diagnostic accuracy of polygraphs for detecting SDB [29,30] and thus, it appears unlikely that polysomnography would have revealed significant different results.

In conclusion, the study results show that postoperative SDB is a frequently occurring phenomenon. It particularly happens when at least expected, in the late postoperative period. At this time patients have been usually transferred to the regular ward. It is conceivable that these SDB’s are a risk factor for postoperative complications, e.g. cardiac arrhythmias, hypertension, delirium, mood disorders and respiratory complications. Careful screening for suspect signs of SDB might contribute to the identification of patients at risk for postoperative complications.

## Competing interests

The authors specifically do not have any financial conflict of interest related to the sponsor of this study, Heinen + Löwenstein, Bad Ems, Germany. The authors declare that they have no competing interests.

## Authors’ contributions

JR: study design, data acquisition and analysis, drafted the article. PS: Critical revision for intellectual content, final approval of the article. SH: Study design, critical revision of the article, final approval of the article. TB: Analysis and data interpretation, final approval of the article. MP: Critical revision for intellectual content, final approval of the article. RC: Acquisition of data, analyzed and interpreted data, final approval of the article. AW: Contributed to concept and design, final approval of the article.
